# A Review of Deep Learning Approaches Based on Segment Anything Model for Medical Image Segmentation

**DOI:** 10.3390/bioengineering12121312

**Published:** 2025-11-29

**Authors:** Dina Koishiyeva, Dinargul Mukhammejanova, Jeong Won Kang, Assel Mukasheva

**Affiliations:** 1 School of Information Technology and Engineering, Kazakh-British Technical University, Almaty 050000, Kazakhstan; koishiyeva.dina@gmail.com; 2 Department of Artificial Intelligence and Big Data, Kazakh National University Named After Al-Farabi, Almaty 050040, Kazakhstan; m.dinargul.14@gmail.com; 3 Department of Transportation System Engineering, Korea National University of Transportation, Uiwang 27469, Republic of Korea

**Keywords:** segment anything model, medical segmentation, hybrid architectures, tuning, domain adaptation

## Abstract

Medical image segmentation has undergone significant changes in recent years, mainly due to the development of base models. The introduction of the Segment Anything Model (SAM) represents a major shift from task-specific architectures to universal architectures. This review discusses the adaptation of SAM in medical visualisation, focusing on three primary domains. Firstly, multimodal fusion frameworks implement semantic alignment of heterogeneous visual methods. Secondly, volumetric extensions transition from slice-based processing to native 3D spatial reasoning with architectures such as SAM3D, ProtoSAM-3D, and VISTA3D. Thirdly, uncertainty-aware architectures integrate probabilistic calibration for clinical interpretability, as illustrated by the SAM-U and E-Bayes SAM models. A comparative analysis reveals that SAM derivatives with effective parameters achieve Dice coefficients of 81–95%, while concomitantly reducing annotation requirements by 56–73%. Future research directions include incorporating adaptive domain hints, Bayesian self-correction mechanisms, and unified volumetric frameworks to enable autonomous generalisation across diverse medical imaging contexts.

## 1. Introduction

The exponential growth in the volume of medical imaging data, coupled with the constant demand for accurate visualisation of pathological structures, has led to an increasing reliance on deep learning-based segmentation methods in medical image analysis [1]. These paradigms have fundamentally redefined the methodological foundations of computer diagnostics by enabling the extraction of complex spatial representations [2]. Segmentation of medical images is a methodological process of assigning a specific label to each pixel of an image to transform the original visual data into a coherent structural representation, highlighting areas of diagnostic significance [3]. Manual segmentation of pathological formations is associated with several objective limitations. The shortage of qualified specialists, combined with the subjective nature of visual interpretation of medical images, leads to significant variability in the determination of tumour structure boundaries [4]. This issue is particularly pertinent when analysing neoplasms with low contrast relative to the surrounding tissue, or when pathological structures resemble typical anatomical structures morphologically [5]. Traditional segmentation methodologies, including thresholding, regional clustering, and atlas registration, have shown high sensitivity to noise, contrast heterogeneity, and morphological variability, thereby limiting their applicability across disparate anatomical locations [6]. Nevertheless, the intricacies that enable deep learning (DL) models to discern intricate dependencies simultaneously render their behaviour inherently erratic, as marginal alterations in data composition and parameter initialisation can precipitate unforeseeable inconsistencies that compromise precision [7]. Although DL methods have improved segmentation accuracy, their reliance on large-scale annotated datasets and the need for retraining in specific domains are two of their main limitations. Furthermore, the high computational load of end-to-end retraining of these architectures limits their applicability [8]. Recent research has focused on basic models trained on large, heterogeneous image corpora, enabling segmentation with minimal preprocessing. The Segment Anything Model (SAM) [9] is presented as a methodology that has shifted the paradigm from task-specific networks to universal segmentation frameworks based on transformer-based visual tokenization and multimodal prompts.

This review examines SAM-based architectures for medical image segmentation across four main parameters: rapid adaptation strategies, multimodal fusion structures, volumetric extensions, and uncertainty quantification mechanisms.

## 2. Background

Medical image segmentation involves converting raw imaging data into a structured form to inform therapeutic decisions [10]. Semantic segmentation consists of assigning a class label to each pixel in an image to identify the type of anatomical structure present [11]. Instance segmentation solves a more difficult task by allowing multiple instances of the same object to be detected in a single image [12]. Efforts to formalise this transformation have long aimed to reconcile the irregularity of biological structures with the analytical rigidity of computational models. Early attempts to impose structure on images were based on contrast and gradients. They treated segmentation as a boundary-detection task based on the physics of image formation rather than the semantics of anatomy [13]. The evolution of segmentation approaches has progressed from traditional computer vision algorithms to modern deep learning architectures [14]. Each of these approaches offers a solution to the fundamental limitations of its predecessor.

Traditional segmentation methods, which have dominated medical imaging for decades, are classified into four main categories depending on the segmentation strategies employed [13]. Intensity-based methods, including threshold segmentation, assume that brightness values are homogeneous within target structures [15]. In contrast, boundary-based methods, such as graph cuts and level sets, focus on detecting sharp intensity transitions as indicators of anatomical boundaries [16]. Regional approaches, such as region growth and clustering methods, aim to combine spatially contiguous elements based on similarity criteria [17,18]. While these methods maintained computational efficiency, they demonstrated a fundamental inability to process morphologically complex anatomical configurations [19]. The necessity for extensive manual parameterisation, coupled with sensitivity to variations in imaging protocols, posed a systemic challenge to clinical integration [20]. The absence of mechanisms to adapt to morphological heterogeneity meant that algorithmic solutions could not be generalised beyond the specified experimental conditions.

The advent of convolutional neural networks has transformed the concept of segmentation, replacing manual feature extraction with hierarchical, data-driven learning [21]. Anatomical variability, as encoded in these methods, was implicitly incorporated into the hierarchies without the need for manual specification. The introduction of fully convolutional networks removed the restriction of fixed input sizes [22]. Dense pixel prediction became feasible by preserving spatial resolution throughout the entire network.

The U-Net architecture, with its symmetric encoder–decoder structure complemented by skip connections, has set a precedent for medical image segmentation [23]. The contracting path in these models gradually reduced spatial dimensions while simultaneously expanding characteristic channels, thereby creating increasingly abstract representations of anatomical content [24]. Conversely, the extensive approach restored spatial resolution via upscaling, but this process alone proved inadequate to recover the fine-grained localization information lost during downscaling [25]. The architectural innovation involved connecting the feature maps from the corresponding encoder layers directly to the decoder layers via pass-through connections that bypassed the representation bottleneck [26]. The reintegration of high-resolution spatial information from deeper layers, combined with semantically enriched contextual features from shallower layers, eliminated the inherent trade-off between localization accuracy and contextual understanding that had been present in previous architectures [27]. The symmetric topology, in the absence of fully connected layers, enabled the processing of inputs of arbitrary dimensions while maintaining dense predictions over the spatial domain [28].

Attention mechanisms introduced the concept of selective weighting of feature representations, prioritising salient areas while suppressing irrelevant contextual information [29,30]. The integration of self-attention operations in transformer-based architectures overcame the limitation of convolutional locality by computing pairwise relationships across all spatial positions [31,32]. Aggregating the global context proved possible regardless of the spatial proximity of elements, thus eliminating the fundamental limitation of receptive fields in convolutional operations.

Despite the architectural intricacy and empirical efficacy of DL approaches, their clinical implementation has remained constrained by the structural limitations inherent in supervised learning paradigms [33]. The intensive training scheme required the use of extensive annotated corpora, and annotating medical images is a particularly resource-intensive undertaking [34]. Models trained with acquisition protocols specific to particular institutions demonstrated reduced performance when confronted with variations in data distribution caused by scanner heterogeneity [35]. This domain-adaptation issue revealed the fragility of learned representations, which lacked the robustness necessary for generalization in the heterogeneous clinical practice environment [36]. The specific nature of supervised learning exacerbated these limitations, as it required the development of independent models with **specialized** annotations for each anatomical target [37].

These systemic limitations prompted a conceptual shift towards fundamental models, moving away from task-oriented control to develop generalizable representations that could be transferred to subsequent tasks [38]. The basic model paradigm segregated representation learning from the iterative vision–language representation learning framework, thereby reducing annotation requirements by acquiring widely applicable visual prior knowledge [39]. This architectural philosophy aligned with the clinical need for adaptable systems that can be deployed across diverse anatomical contexts without exhaustive retraining. 

**Figure 1:**
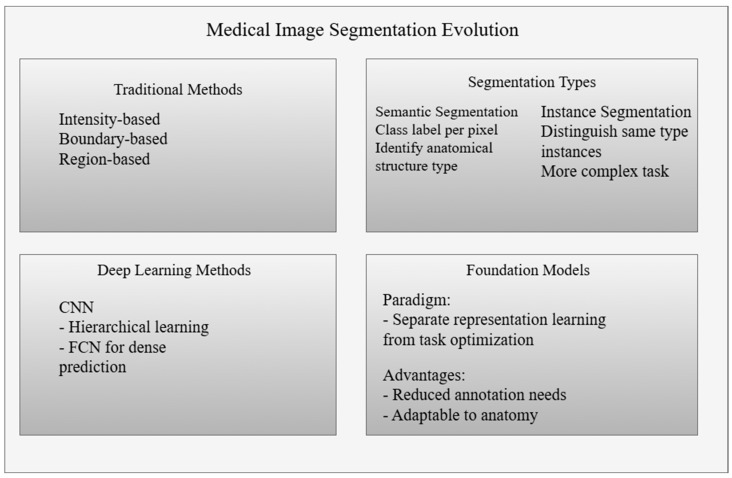
Evolution of medical image segmentation paradigms.

Figure 1 shows a diagram of the evolution of medical image segmentation paradigms. The diagram illustrates the shift from conventional threshold- and boundary-based methods to deep learning architectures and fundamental models. While traditional approaches relied on contrast and manual tuning, deep learning methods introduced hierarchical feature learning, albeit with the limitation of requiring annotations. Fundamental models form universal representations of data portability.

## 3. Search and Selection Strategy

The literature search methodology for this review of SAM-based medical image segmentation followed a systematic protocol. The search strategy covered several scientific databases, including Google Scholar, IEEE Xplore, PubMed Central (PMC), and Scopus, with targeted query formulations specific to the application of SAM in medical imaging. The search captured publications from January 2023 to October 2025, consistent with the emergence of SAM-based architectures. The selection of studies was conducted in several stages: initial database search, selection by title and abstract, full-text evaluation, and final decision on inclusion. The selection was conducted using a procedure based on PRISMA. Two reviewers independently assessed titles, abstracts, and full texts, and disagreements were resolved through discussion. Predefined selection criteria regarding publication type, methodological approach, and technical implementation were applied at each stage. Exclusion criteria excluded works that did not pass expert evaluation, studies without quantitative validation, and publications without explicit implementation of SAM architecture. Data extraction focused on key methodological parameters: dataset characteristics, architecture modifications, training procedures, and performance metrics.

The formulation of search queries was structured into several semantic groups. The main queries focused on the intersection of SAM and clinical imaging, for example, ‘Segment Anything’ AND SAM AND ‘medical imaging’ AND ‘multimodal fusion’ AND segmentation. To identify conceptual, architectural, and prospective studies, broader terms were used, including (‘Segment Anything Model’ OR “SAM” OR ‘SAM-based’ OR ‘foundation segmentation model’ OR ‘prompt-based segmentation’ OR ‘Segment Anything for medical imaging’ OR ‘MedSAM’ OR ‘SAM-Med2D’ OR ‘SAM-2’ OR ‘adaptive SAM’) AND (‘future directions’ OR ‘research prospects’ OR ‘next generation’ OR ‘forecast’ OR ‘open tasks’ OR ‘limitations’ OR ‘tasks and opportunities’ OR “roadmap” OR ‘beyond SAM’ OR ‘post-SAM architectures’). A separate group of queries focused on parameter tuning and was formulated as follows (‘Segment Anything’ OR SAM OR MedSAM OR ‘basic segmentation model’ OR ‘hint-based segmentation’) AND (‘effective parameter tuning’ OR “LoRA” OR ‘adapter tuning’ OR ‘prefix tuning’ OR ‘visual hint tuning’ OR ‘effective fine-tuning’ OR ‘PEFT’ OR ‘BitFit’ OR ‘model compression’ OR ‘knowledge distillation’ OR ‘quantisation’ OR ‘MobileSAM’ OR ‘TinySAM’ OR “FastSAM” OR ‘E-BayesSAM’ OR ‘MoE-SAM’ OR ‘SR-SAM’ OR ‘MedSAM2’ OR ‘AFTer-SAM’ OR ‘computational efficiency’ OR ‘inference speed’ OR ‘FLOP reduction’ OR ‘memory optimisation’ OR ‘training efficiency’ OR ‘real-time segmentation’). The inclusion criteria required that the study present original experiments on the application of SAM or SAM-based architectures, provide quantitative metrics such as Dice, describe the hinting strategy, and provide algorithmic details for evaluating model adaptation. Only medical imaging methods were considered.

The study included works presenting original research on the use of SAM or its derivatives for medical image segmentation, providing quantitative performance indicators such as the Dice coefficient. The medical imaging methods covered included computed tomography, magnetic resonance imaging, ultrasound, radiography, digital pathology and optical coherence tomography. The exclusion criteria were as follows: non-peer-reviewed preprints; review articles without original experiments; non-medical applications; and duplicate publications and studies. In the first stage, 214 publications containing key terms related to the use of SAM and its derivatives in medical segmentation tasks were identified. After removing duplicate records, a sample of 198 unique studies was formed. Screening the titles and abstracts for thematic relevance reduced this set to 124 articles, which were analysed in full. The final sample included 74 studies that met the inclusion criteria.

The heterogeneity of the studies was predictable when synthesising the final sources. The included works differ in terms of **visualization** methods, anatomical targets, dataset size, prompt design, and also the choice of base models. In numerous experiments, SAM-based methods are interactive, while base models are fully automatic. SAM-based methods typically rely on interactive prompts in the form of dots or rectangles, whereas basic models are fully automatic, creating an asymmetry of interaction. A further imbalance in the literature is also apparent. The majority of studies focus on polyps, brain and heart structures, and digital pathology, while relatively few studies address underrepresented organs and rare diseases. Finally, publication bias cannot be ruled out, as most available reports show positive or near-state-of-the-art results for SAM-based architectures.

## 4. Segment Anything Model

The evolution of segmentation represents a transition from functionally closed architectures to a universal model, in which the act of image segmentation becomes a context-dependent form of interpretation. The zero-shot paradigm supports the notion of autonomous extraction of invariant structures of the visual world beyond given classes, and the SAM concept represents the ultimate expression of this universalisation [40]. The SAM method represents this conceptual shift through a three-component architecture in which segmentation does not arise from predefined categorical taxonomies, but from interactive specification of intent [41]. The hint-based segmentation paradigm reframes the segmentation task as a dialogue between user intent and model interpretation, where semantic hints serve as query mechanisms that interrogate a learned visual ontology model [42]. This modality of interaction transcends the conventional boundaries imposed by rigid class and label mappings in controlled approaches, thereby establishing a flexible semantic space in which segmentation boundaries are negotiated through the interaction of query specificity [43].

The SAM constitutes the initial foundation model for universal segmentation, having been pre-trained on the SA-1B dataset, comprising 1.1 billion masks across 11 million images [44]. The architectural topology implements an asymmetric distribution of computational load, with the MAE-pretrained Vision Transformer generating reusable image embeddings. At the same time, a lightweight prompt encoder transforms heterogeneous modalities through positional encoding and CLIP embeddings [45].

**Figure 2:**
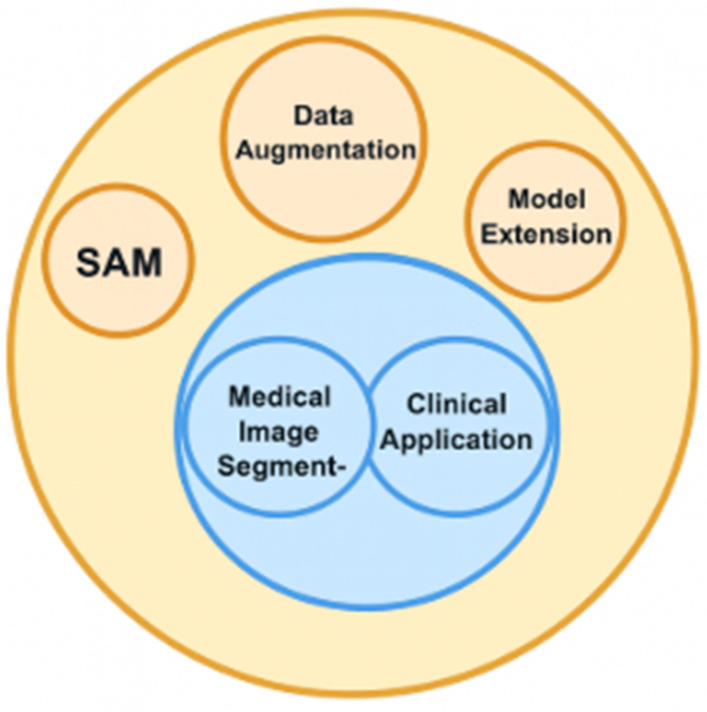
SAM adaptation system for medical applications.

The initial applications of SAM extended beyond the domain of natural images to encompass a range of specialised fields. The analysis of these early adaptations is conceptually valuable for understanding the model’s fundamental limitations and capabilities when confronted with visual characteristics that differ from the training distribution. The ensuing discourse on medical adaptations is contingent upon the establishment of this foundational context. As demonstrated in Ref. [46], the transferability of SAM representations extends beyond their original visual contexts, where their capacity to self-organise interpretations of structures is retained even under conditions of altered spatial statistics. According to the findings reported in Ref. [47], universal SAM generalization mechanisms exhibit resilience to cross-platform variations. In Ref. [48], the model performed invariant transfer of semantic representations between heterogeneous domains of observational data. The initial SAM evaluation of seabed mapping on the multisensor dataset demonstrated the feasibility of AI-driven segmentation in underwater remote sensing, regardless of the measurement technology used [48]. A methodical investigation into the performance of SAM across a range of application domains has exposed heterogeneity in effectiveness, contingent on the particular characteristics of the visual domain. This research has both substantiated the merits of the foundation model approach and elucidated the systematic constraints encountered when confronted with domain-specific patterns [49]. SAM adaptation for medical imaging combines three strategies in Figure 2, universal base representations, style-based data augmentation, and architectural extensions.

The extension of SAM to 3D segmentation was achieved by integrating radiance fields as a connecting prior between multi-view images and spatial geometry. The SA3D approach involves the iterative refinement of 3D masks, alternating between inverse rendering of masks and cross-view self-prompting [50]. It is vital to acknowledge the implementation of additional adaptation in the semantic segmentation of point clouds, achieved using the SAMNet++ hybrid architecture. This architecture sequentially integrates uncontrolled preliminary segmentation using SAM with controlled refinement using a modified PointNet++ [51]. In recent works, the Matcher architecture has enhanced contextual segmentation by leveraging multiple base vision models, implementing bidirectional matching strategies, and fast sampling mechanisms with variable semantic granularity [52]. Recent advancements in the field indicate a gradual evolution of SAM from a vision-based foundation model to domain-invariant segmentation architecture. This cumulative trajectory establishes the epistemic prerequisites for subsequent adaptation to medical imaging.

## 5. Adaptation Strategies of SAM for Medical Imaging

The adaptation of SAM to medical imaging does not signify a straightforward transfer, but rather an interpretative process of epistemic translation that involves recalibrating universal visual priors to the phenomenon of biomedical image formation. The transition from the universal segmentation paradigm of SAM to specialised medical applications reveals a fundamental contradiction between the generalising ability of foundation models and the specificity of biomedical visual patterns. Medical imaging is an epistemologically unique domain in which morphological structures are encoded through modality-specific image formation artefacts, creating a semantic space that is fundamentally different from the natural images in the SA-1B dataset [53]. The strategic reconfiguration of SAM for medical applications is developing along three methodological vectors: a rethinking of prompt mechanisms through clinical descriptors, architectural differentiation based on the physical principles of different modalities, and incorporation of the temporal dynamics of pathological processes [54]. The prompt paradigm is transitioning from geometric pointers to semantically rich anatomical markers. The evolution of the prompt paradigm has reached a qualitatively new level through the automation of prompt generation, as demonstrated by the AutoSAMUS architecture [55]. In this architecture, an auto-prompt generator replaces manual prompt coding, transforming semi-automatic segmentation into a fully autonomous end-to-end process, especially in ultrasound imaging, which is highly operator-dependent. Another research direction exploits the potential of text-driven segmentation, in which natural language clinical descriptors are transformed into visual prompts via multimodal mechanisms, as demonstrated by LuGSAM for chest radiography [56]. The further development of the text-driven paradigm addresses the fundamental problem of semantic complexity in medical descriptors. The study of multi-text joint prompts has been demonstrated to facilitate a strategy for decomposing and recomposing clinical narratives. It has been observed that complex medical descriptions, which integrate anatomical characteristics, imaging modality, and diagnostic priorities, are broken down into atomic semantic units. These units are then optimally recombined through cross-attention mechanisms [57]. A substantial array of studies has evidenced the efficacy of integrating detection models with universal segmenters. To illustrate, combining YOLO with SAM enables automated generation of spatial prompts, thereby ensuring stable segmentation of lung fields despite pronounced intersensor variability [58]. In the context of research [59], the convergence of parallel extraction branches with CLIP-controlled prompts in the PSAM architecture has been demonstrated to be an effective method of multimodal knowledge injection. This is achieved by compensating for the shortage of medical training data through the synergy of semantic prompts and visual adapters in a single computational flow.

The evolution of visual prompts has reached a new level of complexity in the DVPT architecture, which integrates dual mechanisms of local and global modulation [60]. Fine-grained anatomical details extracted by LFPT synergistically interact with the GGP encoder’s noise-suppressing capabilities, forming a robust multi-scale representation of medical structures. The minimalist philosophy of adaptation is evident in Med-SA through the surgical precision of architectural modifications. The model contains 13 million trainable parameters out of a total of 600 million, with the capacity to orchestrate domain transformation. It utilises a Space-Depth Transpose mechanism and a Hyper-Prompting mechanism that provides task-specific conditioning without requiring global retraining. A different approach to the automation of prompts is realised by utilising conventional segmentation models to generate prompts for SAM. Research on U-Net repurposing demonstrates the effectiveness of a two-stage strategy: the initial segmentation obtained by the classical architecture is converted into a set of point or bounding prompts for subsequent refinement through SAM [61]. A salient issue with this approach concerns the processing of erroneous predictions within individual slices, a problem that is addressed by analysing inter-slice consistency and automatically replacing outliers. Despite the growing success of SAM-based frameworks in clinical imaging methods, their transfer to the microscopic domain reveals fundamental limitations of universal a priori segmentations. As shown in the study by Wu et al. [62], zero-shot SAM achieves decent performance in delineating large, contiguous tissue structures in whole-slide pathological imaging. Still, its accuracy deteriorates when confronted with dense cellular architecture and overlapping nuclei. The SAM-Path model proposed in Ref. [63] illustrates this methodological transition by introducing trainable hints at the class level alongside a pathology-specific encoder. The dual adaptation in Ref. [64] enables the model to acquire semantic understanding of histological structures, thus eliminating dependence on manual cues and improving segmentation accuracy in publicly available benchmark tests.

The solution to the problem of prompt scalability in histopathology is Segment Any Nuclei (SAN), which uses an auto-prompting support network to automatically generate thousands of point prompts for densely distributed cell nuclei [65]. This overcomes the fundamental limitation of SAM in processing multiple small objects and demonstrates superiority over fully supervised methods. The implementation of a two-stage strategy for the segmentation of pathological images has demonstrated the efficacy of a cascaded architecture in the processing of multi-stained cell preparations [66]. The development of the μSAM framework has exposed a fundamental contradiction between universality and specialisation in microscopic segmentation. Despite SAM’s initial capabilities, high-quality analysis of microscopic images requires extensive model retraining to account for the physical properties of light and electron microscopy [67]. The integration of adapted models into a unified software environment creates a practical toolkit. However, researchers have noted the need for separate training for different types of microscopies, pointing to the limitations of current foundation models in capturing the full spectrum of biomedical visual modalities. A comparable study has shown that integrating task-specific and foundation models **via** the X-Gated Fusion Block demonstrates the effectiveness of hybrid architectures. This is achieved by combining local features of the U-Net encoder with global representations of SAM through gated attention mechanisms, thereby improving the Dice metric relative to isolated approaches. The preservation of nuclear structure detail when using contextual information from the foundation model is enabled by adaptive modulation of contributions from different feature sources [67]. As asserted by Archit et al. [67], a meticulously calibrated version of SAM, in conjunction with a watershed algorithm, yields substantial advancements in the field of mitochondrial segmentation in electron microscope images, as demonstrated by. The efficacy of this approach has been proven to achieve precise separation of mitochondrial structures while maintaining congruence between local gradient contours and global representations, thereby enhancing the precision and reliability of the segmentation process. The paper by Wang et al. [68] presents the SegAnyPath model, demonstrating the evolution of the foundation segmentation paradigm towards multi-level adaptation: the integration of multiscale proxy learning, distillation across colouring variations, and the modular Mixture of Experts architecture forms a stable representation space that maintains accuracy and consistency across inter-organ and inter-style variations in pathomorphological images. Continuing the line of research aimed at overcoming the limitations of universal segmenters when transferring them to the medical field, the recent study by Faska et al. [69] showed that targeted fine-tuning of SAM on specialized biomedical datasets provides a significant increase in accuracy while maintaining zero generalization ability, forming a transition from universal architectures limited by domain shift to adaptive systems in which the alignment of pre-trained representations with tissue morphology specificity becomes a key condition for model robustness. In a further study, the CellSAM model was presented, in which the concept of universal segmentation is transformed into a cell-oriented architecture: through asymmetric dual encoding and feature distillation of large-scale models, high accuracy in nucleus identification is achieved despite morphological heterogeneity and dense clustering, reflecting further deepening of SAM adaptations into the microstructural space of pathomorphology [70]. As demonstrated in Ref. [71], application of SAM within the SAM-L framework facilitates the transformation of weak annotations into accurate pixel labels through a zero-shot mechanism, underpinned by molecular-driven learning. This process establishes a cognitive space of agreement between visual features and biochemical markers, thereby enabling the annotation process to become independent of expert intervention and acquire the properties of self-organizing interpretation of pathological structures. In the course of a scientific investigation into the automated stratification of renal cell carcinoma (RCC) tumours, the application of the statistical model known as SAM to the segmentation of RCC tumours has been demonstrated to effect a transition from local morphometric analysis to an integrated representation of tumour structure. In this integrated representation, the encoder’s latent features form a joint space with radiomic parameters, thereby ensuring coherent preoperative differentiation of malignancy grades and increased prognostic reliability [72]. A similar study [73] demonstrates the application of SAMCell in cell microscopy, providing a segmentation process with generalized interpretation of morphological structures by aligning the feature spaces of a universal model with the optical properties of cells, ensuring reproducible contour identification without annotation dependence. The strategies for adapting to prompts in a representative way are systematised in Table 1. The methodological innovations and imaging modalities targeted are highlighted.

The corpus of research demonstrates a transition from architectural adaptations of a specific nature to a holistic model of interpretation. In this model, segmentation is regarded as a process of combining visual and contextual representations in a unified cognitive space. The development of principles of multimodal coupling is imperative.

## 6. Multimodal Fusion Strategies in SAM-Based Frameworks

The integration of multimodal data into SAM-based architectures provides a methodological framework for harmonizing diverse visual representations of biomedical objects. The interaction of modalities is interpreted not as data synthesis, but as a reconfiguration of feature spaces to establish their functional equivalence within a single latent field.

This conceptual reformulation interprets multimodal fusion as a process of epistemic alignment rather than a mechanical combination of heterogeneous input data. Recent studies, exemplified by the FusionSAM architecture [74], demonstrate the potential of integrating latent space to build stable correspondences between different visualization methods through vector quantization and cross-attention mechanisms. Such architectures generate semantically coherent fusion cues that guide segmentation with adaptive accuracy across different visual domains. The MSAM model builds on the conceptual foundation laid by FusionSAM, extending the integration principle to the biomedical context by coupling structural and diffusion representations [75]. In this model, modality alignment arises from self-organising interactions in the latent feature space. The model architecture constitutes a dynamic system of mutual adaptation between data sources, where coherence is achieved not by combining them, but by correlating information invariants [75]. The Multi-Modal SAM-adapter study posits that multimodal integration can be conceptualised as a deliberate transformation of latent space, in which the incorporation of additional sensory features does not expand visual representations but rather refines their semantic content [76]. The injection of complementary modalities is selective, ensuring a functional balance between universality and contextual adaptability [76]. Another study attempts to reinterpret knowledge distillation from Segment Anything to the lightweight U-Net architecture not as a simplified parameter inheritance, but as a process of cognitive compression, in which the universal latent structures of the fundamental model are compressed into the adapted contours of a specialised segmenter [77]. The result is an architectural configuration that preserves the heuristic power of the large-scale model in a condensed form, providing comparable accuracy and stability in clinical scenarios with limited resources [77]. In the context of developing advanced prognostic analysis methodologies, the SAMMS architecture is being developed, a deep learning model for predicting the survival of cancer patients [78]. In contradistinction to classical approaches to visual segmentation, this method conceptualizes SAM as a semantic interface that establishes correlative links between the morphological patterns of histological images and a set of molecular-genomic profiles with clinical parameters. In Ref. [79], it was noteworthy that integrating modality-specific and cross-modal computational subnetworks constructs a coherent latent space in which visual descriptors and ohmic data form a unified probabilistic continuum.

In the context of ongoing research endeavours to augment the capabilities of SAM-based models in medical imaging, a particular focus has been on the cross-modal attention method. The combination of local structural information and global semantic representations has been shown to yield a more stable reconstruction of complex biomedical objects. The other study of the multimodal configuration of SAM interprets the process of integrating textual and visual signals as the formation of a unified interpretative space in which linguistic descriptors set the direction for the convergence of local and global features, and the topology of attention is restructured depending on the semantic density of the medical context. In the study by Gao et al. [80], multimodal segmentation of cellular structures is described as a process of integrating heterogeneous representations into a single latent space, in which visual features of cell morphology are correlated with textual descriptors via a system of adaptive projections. In developed architectures, the segmentation module, the multimodal prompt, and the output object block form a closed loop of semantic coordination. Each component of the aforementioned model processes input data, and in the context of intermodal coherence, dynamically refines its meaning. In the MedFocusCLIP model [81], attention is formed as a self-organizing process of internal correlation between spatial and semantic structures. In this process, the segmentation features of SAM2 interact coherently with the CLIP encoder’s latent representations, adjusting their configuration in accordance with the image’s contextual relevance [81]. This internal dynamic brings together the levels of local perception and conceptual differentiation, forming a unified cognitive space. 

In a recent study, multimodal segmentation of stroke lesions has been proposed as a method for harmonising heterogeneous diagnostic signals in a single feature space [82]. The SMF-Net architecture implements this idea through a Siamese encoder based on the Swin Transformer and an adaptive co-attention system that links modality-specific and contextual representations [83]. In the domain of optical coherence tomography angiography, the multimodal concept investigated in the latest study is implemented using the WS-SAM architecture, which combines spatial and frequency features in a dual-domain encoder, thereby providing noise and artefact suppression while preserving vascular details [84]. The integration of a wave fusion module facilitates adaptive alignment of textural and structural components, while self-prompting, guided by prototype learning, directs the model’s attention to microvascular formations. In the context of morphologically complex structures, multimodal interaction manifests itself as a fusion of geometric and semantic priors within a single prompt space. The APPL (Annular Prior Prompt Learning) method is based on a bespoke annular prior encoder that transforms multiple prompt points into a continuous regression feature space. The incorporation of morphological attention into training establishes topological consistency across regions, thereby minimising the effect of intraclass variability [85]. Segmentation of breast tumours within multimodal architectures reveals multilevel coherence between acoustic-visual features of ultrasound imaging and clinical-diagnostic descriptors, including parametric classifications and BI-RADS assessments. A process of semantic transduction unfolds modality interaction. This involves clinical attributes reconfiguring the topology of visual features. The purpose of this reconfiguration is to generate a unified semantic field of interpretation for further segmentation [86].

Multimodal SAM systems [75] form a new ontology of image analysis, according to which the interaction of modalities is viewed as a mutual transformation of semantic spaces. The configuration of features is not the result of data aggregation; instead, it is an internal correlation of structures that maintain the coherence of perception when the observation context changes. In such models, segmentation is understood to emerge as a process of semantic alignment, whereby morphological, textual, and clinical descriptions mutually refine the boundaries of interpretation. SAM fulfils an intermediary function between visual material and diagnostic abstraction, thereby ensuring the continuity of the semantic field.

## 7. Volumetric Extensions of SAM Architectures

Within the expanding paradigm of volumetric models, FastSAM3D functions as a key reimagining of segmentation efficiency, translating the interactive logic of two-dimensional SAMs into a compact volumetric form [87]. A progressive layer-wise distillation mechanism condenses the representation density without disrupting semantic continuity [87]. In the context of the emergence of three-dimensional segmentation paradigms, SAM3D adopts a novel methodological position, interpreting the principle of universal image division as a continuous spatial mapping rather than a set of flat projections [88]. The model deviates from the conventional approach of dividing volume into two-dimensional segments, thereby transforming the analysis process into a comprehensive reconstruction of a volumetric field. In this field, the boundaries of objects become characterised by topological variations in feature density. This transformation of architectural logic establishes a different type of epistemic correspondence between learning and spatial perception: three-dimensionality becomes not just a measurement of data, but a carrier of contextual integrity. Consequently, SAM3D translates the SAM paradigm from the plane of universal applicability to the realm of coherent volumetric reasoning [88]. Shen et al. [89] developed the ProtoSAM-3D model, which implements the concept of volumetric semantic segmentation as a process of stratification of feature spaces, where interactive SAM mechanisms are transformed into prototype-semantic mapping of masks. A transformer that is aware of spatial relationships builds a hierarchical consolidation of CT and MRI signals, forming a continuous latent manifold within which the mask correlates not with a fixed category, but with a dynamically generated prototype. It can be posited that the model’s purpose is to construct a system of mutual correspondences between the morphological and semantic levels, rather than to reproduce the anatomical structure. In three-dimensional medical volumes, the UP-SAM model extends the concept of partially annotated learning, integrating the SAM architecture into a probabilistic adaptation circuit [90]. In this circuit, epistemic and aleatory uncertainties are correlated as complementary stabilisation mechanisms, ensuring consistent extraction of anatomical boundaries and stable alignment of spatial structures with the universal priors of the base model with minimal annotation. In a recent study [91], the 3D-SAutoMed model was developed to systematise three-dimensional perception in the SAM paradigm by integrating local-global attention mechanisms and automated prompt generation. The concept under discussion is based on the interplay between cross-slice and intra-slice coordination. In this paradigm, the exchange of historical information ensures the continuity of the spatial context, and the rectangular prompt generator forms an autonomous initialisation contour without user involvement.

In studies focused on the three-dimensional development of SAM architectures, the SegMamba concept [92] offers an alternative perspective on the spatio-temporal organisation of medical data, wherein a volumetric image manifests as a continuous field of interdependent states. In this paradigm, the structural elements of the scene interact through extended correlations that link local contours with global morphological patterns [92]. The state model implements a mechanism for the sequential propagation of information throughout the volume, highlighting spatial relationships while maintaining computational efficiency. In the context of the development of three-dimensional architectures, the CT-SAM3D model [93] reflects the transition from localised organ-specific solutions to systematic anatomical segmentation of the entire body. In contrast to conventional 2D modifications oriented towards slice-by-slice processing, this approach operates in a continuous three-dimensional space of features. The interaction between prompts and morphological structures forms a coherent field of interpretation in a volumetric context. The principle of topological consistency is realised through the progressive encoding of spatial prompts with inter-patch context linkage. The study developed the 3DMedSAM architecture [94], which implements cross-dimensional adaptation of SAM to volumetric medical data by replacing the patch embedding module with a native recognition module for three-dimensional pattern recognition while preserving frozen pretrained weights. The integration of the 3D Adapter enriches deep features with inter-slice spatial correlations. At the same time, the multi-scale decoder addresses the scale variability of pathological structures, ensuring robust segmentation for both single-target and multi-organ tasks.

**Figure 3:**
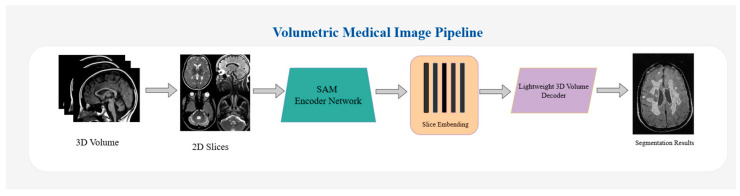
MedSAM adapts the basic model for medical imaging.

Extending the foundation model paradigm to volumetric medical segmentation enables SAM-Med3D [95] to overcome the limitations of task-specific architectures by implementing an entirely learnable 3D structure. The model demonstrates robust zero-shot generalisation across 16 diverse datasets, covering heterogeneous anatomical structures and imaging modalities. Sparse 3D point-based prompting enables accurate delineation of previously unseen target objects [95]. The key issue hindering the practical application of promptable segmentation models in the clinical domain is the need for expert specification of prompts. This process requires considerable time and domain expertise from clinical specialists. AutoProSAM [96] overcomes this limitation by combining automated prompt learning with parameter-efficient adaptation for 3D medical imagery, achieving state-of-the-art performance in multi-organ CT segmentation with no manual prompt generation [96]. In the most recent study, a model was developed and adapted in the ophthalmological context of foundation models. SAM 2 and MedSAM 2 were adapted for volumetric segmentation of retinal biomarkers in optical coherence tomography [97]. Figure 3 shows the architecture of the MedSAM model.

The issue of constrained data availability in volumetric medical imaging is addressed by SAM2Med3D [98], which operationalizes the SAM 2 video foundation model for 3D breast MRI segmentation by conceptualising volumetric scans as temporal sequences. The architecture implements a multi-stage pipeline that integrates a lightweight task-specific network with a foundation model through a novel spatial filtering strategy to identify reliable slices as high-quality prompts. This is in conjunction with a confidence-driven fusion mechanism that mitigates segmentation drift and ensures volumetric consistency [98]. The challenge posed by the incongruence between two-dimensional vision transformers and volumetric medical data is addressed by RefSAM3D [99], which implements a cross-modal reference paradigm by integrating a 3D image adapter with text prompt generation. A hierarchical attention mechanism has been developed to enable multi-scale feature integration, whereby a modified visual encoder performs native 3D processing in conjunction with a retrained mask decoder for direct volumetric prediction.

The VISTA3D architecture developed by He et al. [100] was a paradigm example of such a model, which was initially designed to solve three-dimensional medical imaging tasks. A fundamental aspect of this approach is the integration of automatic segmentation with interactive correction mechanisms, without specialised training for specific tasks. Empirical evidence substantiates the model’s functional capabilities, as demonstrated by its comparable efficiency in processing 127 anatomical structures and solving interactive segmentation-correction tasks. The methodological basis of the present study is the original technique of three-dimensional supervoxels, which provides the transfer of pre-trained two-dimensional representations into three-dimensional space. Within the research trajectory of three-dimensional SAM adaptations, the SAM3X model [101] represents an attempt to systematically rethink the interaction between pre-trained two-dimensional representations and the volumetric structures of medical images. The architecture constitutes a three-axis coding system that employs the original SAM encoder across three spatial directions to generate an isotropic volume representation, thereby facilitating the capture of contextual dependencies between slices [101]. In a similar study, Zheng et al. [102] proposed the PanSAM3D model, which focuses on automated pancreas segmentation in multi-sequence MRI data. The architecture provides a consistent three-dimensional representation of anatomical structures by combining self-attention and triple convolutional attention mechanisms. In Ref. [103], a hybrid prompt encoder combines sparse and dense signals, while a multi-level decoding scheme with a volume-oriented loss function supports accurate contour differentiation. In a related direction, the PPA-SAM model [103] has been developed to focus on three-dimensional segmentation of dental structures from cone beam computed tomography data. The architecture synthesises the generalising capability of SAM with the local accuracy of convolutional networks through a dual encoder. Within the adversarial training loop, the three-layer convolutional subnetwork operates concurrently as both a discriminator and a generator [103]. A recent study aimed to transfer fundamental visual models to dental diagnostics by proposing the adaptation of SAM2 for tooth segmentation in panoramic X-ray images [104]. This approach involves transitioning from natural images to medical images by introducing adapter modules that reconfigure spatial-semantic representations to suit the characteristics of X-ray visualisation. Built-in ScConv blocks and controlled attention mechanisms enhance the model’s ability to extract multi-level features in low-contrast conditions. In the development of SAM-oriented architectures, one direction is represented by the SAM-Guided U-Net model [105]. Here, the semantic field of the fundamental model acts as a coherent regulator of cross-scale representations. Transformer blocks establish global structural dependencies within lung volumes, and a modified 3D U-Net extracts small-scale textural features to ensure the topological integrity of node boundaries [103]. The mutual coordination of these levels of representation generates spatial-semantic unity.

In essence, the evolution of volumetric SAM architectures reveals a gradual transition from strategies designed to overcome dimensional discrepancies to all-encompassing frameworks that incorporate various segmentation methodologies within a unified architectural framework. The trajectory of development, from slice-wise processing to native three-dimensional feature extraction and unified automatic-interactive systems, illuminates the fundamental tension between computational tractability and semantic fidelity in volumetric medical segmentation. The variety of architectural solutions suggests the absence of consensus design principles for three-dimensional foundation models, underscoring the need for task-adaptive architectural selection rather than universalist frameworks. Nevertheless, cumulative empirical evidence confirms that volumetric SAM adaptations are a viable alternative to traditional, task-specific architectures, offering the potential to reduce annotation burden and extend cross-domain transferability substantially. In 3D variants including SAM3D [88] and ProtoSAM-3D [89], volumetric processing is based on a modified ViT encoder featuring axial attention, as well as lightweight 3D CNN decoders. However, these models require a large amount of GPU memory and are sensitive to anisotropic distance, often resulting in degraded boundaries in the axial dimension.

## 8. Reliability and Uncertainty

In the process of implementing segmentation models in clinical practice, a fundamental question arises regarding the reliability of the results. Clinical testing of segmentation models reveals problems arising from distributional shifts caused by the heterogeneity of medical data. This review summarizes the methodological principles for verifying prognostic reliability. Zheng et al. [102] proposes a conceptual extension of SAM, in which the model automatically identifies explainable semantic areas that serve as a basis for interpreting neural network decisions. The method is based on the per-input equivalent (PIE) scheme [106]. The FastSAM-3DSlicer study [107] is developing a technological environment that integrates SAM models into the clinical workflow via an extension for 3D Slicer, aiming to strengthen the reliability and reproducibility of SAM architectures. As part of this implementation, the interactive processing of two- and three-dimensional medical volumes is coupled with an uncertainty assessment mechanism [107]. A similar study has developed a conceptual framework for assessing regional uncertainty, correlating annotation quality with the topology of the fuzzy boundaries of organic structures [108]. Training is organised as a stratified process in which samples are distributed by label reliability, and signals are transmitted sequentially to less confident areas, forming a confidence gradient within the training space. In this system, the boundary-oriented module regulates semantic stability by calibrating local probability fluctuations and maintaining consistent segmentation decisions in the presence of annotation noise and incomplete data.

Wei et al. [109] developed the I-MedSAM reliability concept, implemented by transitioning from discrete to continuous segmentation representations, ensuring topological stability of boundaries and consistent predictions across spatial resolutions. The architecture uses an adapter to enrich SAM features with high-frequency components and implements an Implicit Neural Representation (INR) as a continuous decoder that produces a dense probability field rather than a pixel map. An uncertainty-driven sampling strategy is integrated into the training. This method allows optimization to be directed to areas of low model confidence, reducing the likelihood of systematic errors. Zhang et al. [110] laid the methodological foundation for the UR-SAM block, which is formed around the principle of using uncertainty as an active self-correction mechanism in automated segmentation. It combines a localized auto-prompting system with a stochastic input signal expansion module, creating an ensemble of prompts that reflect the model’s internal confidence distribution. Subsequently, the uncertainty estimates are integrated into the spatial refinement process, in which rectification is achieved through adaptive redistribution of weights between confident and ambivalent areas.

In the novel conceptual design of Cascaded Diffusion–SAM, the interaction between generative and segmentation contours is not linear. Instead, it unfolds as a process of mutual correction of semantic fields [111]. The diffusion model produces a probabilistic image space in which uncertainty signals divergence between the reconstructed and pathologically distorted structures. These fluctuations generate prompts that, when fed into the SAM model, do not directly refine the image; instead, they set the trajectory of attention redistribution within the hidden feature space. In the Uncertainty-Guided Cross Attention module, this redistribution takes the form of semantic resonance, whereby the noisy areas of the diffusion layer are converted into informative increments of segmental perception. In recent study [112] on muscle structure segmentation in neuromuscular disorders, SAM is introduced as a means of balancing computational accuracy and epistemic reliability, rather than as an alternative to existing architectures. In the encoder–decoder configuration, fine-tuning the model improves the definition of fascicular structures. However, Han et al. [108] also introduced a new metric, ‘topology of confidence’, in which error is interpreted as statistical density. In this configuration, the model is not trained to recognise muscle fibres directly, but rather to calibrate a probability space in which confidence becomes a function of the pathological context.

Deng et al. [113] developed the SAM-U model in their study, which is an approach to improving segmentation reliability. This model integrates uncertainty into the very architecture of the computational process. Using multi-box prompts creates a stochastic prediction space, enabling evaluation of the model’s variability via an ensemble of independent samples. Applying the Monte Carlo strategy and parameterising prior distributions establishes a mechanism for calibrating internal reliability, providing a quantitative assessment of confidence at the pixel mask level. This principle of recursive self-assessment transforms SAM into a system inseparably linking segmentation and its epistemic verification. In the subsequent stage of the evolution of SAM-oriented models, uncertainty began to be viewed as a computationally controllable parameter. Thus, the P^2^SAM model [114] proposes a probabilistic extension of the prompt mechanism, as in previous studies, in which the latent space of SAM is redefined as a stochastic domain for generating masks. This architecture transforms the model’s sensitivity to prompts into a tool for modelling ambiguous boundaries and discrepancies in expert annotations [114]. The application of probabilistic prompt encoding forms a robust mechanism for generating a set of probable segmentations in conditions of limited annotated samples, transitioning to models focused on interpretability, probabilistic consistency, and statistical calibration of medical decisions.

In the recent work [115], the EviPrompt method is a zero-shot prompt generation strategy based on an evidential approach. The model uses a single ‘image-annotation’ pair as a reference to generate prompts by comparing the features of the reference and target images. The system incorporates both a committee voting mechanism and in-context inference, enabling the model to adapt to the medical domain without additional training. In the recent results focused on reducing reliance on full expert annotations, a weakly supervised segmentation scheme for intracranial haemorrhages is presented, combining a YOLO detector with a modified SAM architecture and uncertainty correction [116]. The mechanism combines lesion boundary detection with subsequent segmentation, using YOLO-predicted bounding boxes as a source of automatically generated point prompts, enabling adaptation to weak annotations. Integrating the uncertainty rectification procedure calibrates the model’s confidence without requiring full masks. Within the ongoing research into improving the reliability and interpretability of fundamental segmentation models, the U-MedSAM [117] architecture has been developed as a synthesis of MedSAM and an uncertainty accounting mechanism, incorporating Sharpness-Aware Minimisation (SharpMin) optimisation. By integrating the regional, distributional, and pixel components of the loss function into a single adaptive system [117], this approach enables each component to regulate confidence across different levels of spatial heterogeneity. This structure provides consistent uncertainty modelling, treating contour variations and prediction fluctuations as a source of corrective information rather than noise.

The recently developed E-BayesSAM [118] architecture is designed as an integrative circuit in which Token-wise Variational Bayesian Inference (T-VBI) and the Self-Optimising Kolmogorov–Arnold Network (SO-KAN) do not function as isolated modules, but rather as mutually regulating mechanisms within a single, probabilistically interpretable system. Within T-VBI, SAM output tokens are mapped to a space of latent stochastic variables, enabling dynamic Bayesian uncertainty estimation without additional training. This shifts the emphasis from classical regularisation to probabilistic parameter self-correction. SO-KAN interprets the results and rebuilds the feature structure through self-learning spline activations, minimising redundant dependencies and identifying topologically stable decision-making tokens. The interaction between these components results in a hierarchy of consistent probabilistic representations. Yao et al. [119] developed the FNPC-SAM methodology, in which uncertainty is introduced not as a confidence metric but as a control element that corrects model errors when working with noisy medical images. The algorithm employs multi-window hint augmentation during inference, where variations in input queries yield an ensemble of probabilistic masks. Based on the discrepancies in these masks, a procedure for correcting false-positive and false-negative areas is developed, based on the assessment of aleatory uncertainty. This procedure operates independently of retraining and directly modifies the structure of the predicted masks, minimising erroneous regions. An additional Single-Slice-to-Volume (SS2V) module extends this principle to three-dimensional tasks by enabling volume reconstruction from a single annotated projection. The evolution of uncertainty quantification in SAM-based architectures reflects a conceptual shift from evaluative metrics to architecturally integrated mechanisms of probabilistic self-correction, which are a key part of this shift. Table 2 summarises representative uncertainty-aware SAM frameworks, highlighting their methodological strategies and key innovations.

The methodological principles of adapting SAM architectures for medical segmentation reveal a conceptual transformation in the understanding of uncertainty. There is a shift from perceiving it as a purely evaluative metric to an architecturally integrated mechanism of probabilistic self-correction. The evolutionary path from ensemble approaches to evidence-based and Bayesian paradigms reflects the formation of requirements for clinically interpretable assessments. The methodological foundation for clinically applicable SAM methodologies is formed by integrating quantitative uncertainty assessment via variational inference, probabilistic prompt encoding, and continuous implicit representations.

## 9. Comparative Analysis of SAM Architectures for Medical Image Segmentation

To evaluate the effectiveness of SAM-based architectures in the broader context of medical image segmentation, it is necessary to compare their quantitative and qualitative results with those of the current best-performing deep learning methods. The quantitative performance evaluation was based on standard medical segmentation metrics [120]. The Dice coefficient measures the degree of overlap between the predicted mask and the expert annotation. Intersection over Union (IoU) evaluates the accuracy of region localization [121].

In a recent experiment, the basic SAM model was considered as the initial architecture, demonstrating high efficiency in brain MRI segmentation on the LGG dataset. When using bounding-box prompts, the model achieves Dice scores of 0.92 and IoU scores of 0.87. The results obtained in Ref. [120] exceed those of traditional convolutional architectures, including DeepLabV3 (Dice 0.81), DenseNet (Dice 0.83), and U-Net (Dice 0.88). The methodological basis of the approach is transfer learning with a frozen SAM encoder. In light of the recent surge in hybrid and multi-stage paradigms, in which basic models are combined with optimized convolution-based models, the work [121] makes a notable contribution with its SMU-Net architecture. The model under consideration evinces a two-stage refinement structure, in which a U-Net with enhanced attention is used to generate coarse structural prior information, as well as a customizable SAM decoder using hierarchical guidance with hints: masks predicted in the first stage are converted into region-bound hints that condition SAM segmentation refinement at the token level. Quantitative evaluation on the Kvasir-SEG dataset [122] demonstrates a Dice coefficient of 0.946, outperforming convolutional architectures by 2–5 percentage points and surpassing the baseline convolutional networks U-Net and U-Net++ by 12.8% and 12.5%, respectively. In a related line of research, Ref. [123] presented the SP-SAM model, developed to overcome the limitations of traditional SAM architectures in medical segmentation scenarios. The proposed structure is distinct from previous fine-tuning approaches, such as MedSAM and SAMed architectures, in that it retains the frozen SAM base and incorporates a self-optimizing prompt mechanism built on two auxiliary components: The following auxiliary components are to be considered: Isolated Noise Removal (INR) and Multi-Point Automatic Prompt (MPAP). Empirical validation of the methodology on heterogeneous medical datasets, Kvasir-SEG and ISIC 2018 [124], demonstrated a statistically significant improvement over the baseline SAM implementation with a modest number of instances, achieving Dice scores of 65.9 and 66.9, respectively. Despite the observed discrepancy with fully supervised solutions in absolute terms, the proposed Self-Prompting SAM architecture demonstrates operational feasibility for sparsely labelled data [125]. The PSF-SAM method [126] addresses catastrophic forgetting in fine-tuning SAM for polyp segmentation by employing a parametrically efficient training strategy. The key architectural innovation is to freeze the initial SAM parameters while introducing trainable tokens of different scales adapted to the morphological variability of polyps. The method’s validation on the Kvasir-SEG and CVC-ClinicDB [127] datasets demonstrates its superiority over standard fine-tuning approaches. The mDice score attains values of 0.9393 and 0.9421, respectively, which surpass the outcomes of full fine-tuning by 2.7–3.4%. In a comparable study [128], the FCSAM model employs a two-branch architecture combining a U-Net for local analysis and a SAM for global context, using the LNLoRA fine-tuning strategy [129]. The validation of Kvasir endoscopic data has been undertaken, with IoU and Dice metrics of 79.3% and 88.1%, respectively. These figures represent an improvement in contour accuracy of 12.7% over baseline SAM implementations, whilst concomitantly reducing the computational load by 34%. The BiSeg-SAM method [130] builds on this approach by implementing an alternative weakly supervised post-processing strategy for medical image segmentation. This strategy combines SAM with a CNN module, enabling the integration of global context and local details. The following architectural innovations are worthy of note: first, the WeakBox module with the MM2B algorithm for automatic prompt generation; second, the DetailRefine module for precise boundary determination. The model’s validation on five datasets of polyps and dermatological images shows comparable performance to SAMU-Net, achieving Dice scores of 0.919 on the Kvasir dataset and 0.904 on ETIS [131].

The issue of domain shift when adapting foundation models to medical tasks necessitates the development of specialised regularisation strategies. The SR-SAM method [132] implements a space regularisation approach by iteratively removing domain-specific directions (TSD) from pre-trained weights, followed by aggregating updates using the EMA LoRA module. Quantitative validation on Kvasir, CVC-ClinicDB, and prostate MRI datasets RUNMC and BMC [133] reveals average Dice scores of 81.46% and 83.64%, respectively. A comparative analysis with baseline PEFT methods demonstrates a 1.1–1.3 percentage point increase in generalization ability while maintaining parametric efficiency.

The model developed in Ref. [134], the WSPolyp-SAM study, implements a weak supervision strategy for polyp segmentation, using limited annotations in the form of bounding boxes to generate SAM pseudo-masks. The proposed architectural solution involves freezing the SAM encoder image whilst fine-tuning the encoder prompt and decoder masks. In this architecture, a three-step process is utilised to enhance the quality of pseudo-masks. This process involves multi-augmentation, mask fusion, pixel entropy weighting, and morphological post-processing. The validation of the model on five medical datasets demonstrated Dice metrics ranging from 0.907 to 0.945, indicating a 56–62% enhancement in performance when compared to complete control, along with a 70–73% reduction in annotation time. The ColonDB dataset [135] shows the largest gain, with a 9.4% increase in Dice score relative to fully controlled analogues. The method is subject to certain limitations, including the dependence of pseudo-mask quality on the quality of the initial SAM segmentations. The model presented by Liu et al. [136], LuGSAM, is a framework that integrates text prompts via Grounding DINO and SAM for X-ray segmentation. The model achieves a Dice score of 0.97 for the right lung with ViT-h, outperforming baseline implementations by 12.3% on the NIH CXR and Montgomery County datasets [137,138]. Iterative bounding box correction improves detection accuracy by 15.7%, and text prompts such as “right lung” achieve a confidence score of 0.58. There is also an 18.9% improvement in segmentation, as measured by the IoU metric, compared to standard SAM. The recently proposed methodology, MedCLIP-SAM [139], incorporates the BiomedCLIP model [140], which has been retrained with SAM to segment medical images using text prompts. The efficacy of the proposed methodology is evident in its performance on the BUSI (breast ultrasound) [141], Brain Tumour MRI, and COVID-QU-Ex (lung X-ray) datasets [142], yielding Dice scores of 0.856, 0.881, and 0.913, respectively. The application of weak supervision has been demonstrated to enhance lung segmentation by 4.2%. The proposed approach shows superior tumour segmentation performance compared with the fully supervised ResUNet, yet it exhibits suboptimal lung segmentation performance. Cross-modal search on the ROCO dataset confirms the DHN-NCE loss’s advantage, achieving an accuracy of 84.7%.

Expanding on the functionality of the basic SAM-2, the TGSAM-2 architecture [143] introduces text-controlled segmentation mechanisms through a module that aligns semantic descriptors with visual features and a memory encoder for spatiotemporal tracking in medical image sequences. Validation on ACDC myocardial magnetic resonance imaging [144], MSD Spleen computed tomography [145], prostate ultrasound data [146], and CVC-ClinicDB colonoscopy reveals Dice scores of 87.63%, 89.34%, 92.75%, and 85.10%, respectively. The multi-structure configuration, with simultaneous training on heart anatomical structures, achieves 85.70%, exceeding the performance of point-and-click methods. The transition from two-dimensional to volumetric segmentation requires modifications to architectural components to accommodate spatial dependencies across slices. The AFTer-SAM method [147] adapts the basic SAM model by integrating an axial fusion transformer that simultaneously captures intra-slice and inter-slice information. The architecture retains the pre-trained ViT encoder [148] and adds an axial attention mechanism via parametrically efficient LoRA tuning. Quantitative evaluation on the Thorax-85 and SegTHOR thoracic datasets [149,150] reveals average Dice scores of 92.75% and 92.42%, respectively. The most significant increases in morphologically complex structures were observed, with the oesophagus increasing by 82.34% and the spinal cord increasing by 91.24%. A comparative analysis of AFTer-UNet and MSA reveals consistent improvements of 0.4 to 1.2 percentage points across metrics.

Another notable development is the integration of segmentation and localisation mechanisms within a single architecture. The AutoSAME framework [151] is a specialised system for automated left ventricular measurements. It integrates the SAM segmentation paradigm with the localization of anatomical landmarks, as defined by established clinical protocols. From an architectural standpoint, the method augments the base model with an additional CNN branch for regressing heat maps of key points. The filtered cross-attention module between branches ensures the transfer of frequency-spatial features from the segmentation to the localization subsystem. The spatially-oriented prompt alignment module generates embeddings based on prior anatomical knowledge of the left ventricle geometry. The application of the CAMUS dataset [152] has yielded Dice scores of 0.928 for segmentation and PCK scores of 0.948 for point localization. The correlation between measured volumes and expert estimates ranges from 0.961 to 0.964, while for ejection fraction, it reaches 0.827. As part of the ongoing development of SAM-oriented approaches, the Wu et al. [153] proposed the SAID-Net method, which integrates implicit neural representations (INR) with a SAM architecture tailored for the analysis of echocardiographic sequences. The model incorporates a Hiera encoder, which facilitates multi-level feature extraction, with a Mask Unit Attention Decoder. The use of the CAMUS dataset for validation yielded a Dice of 93.2% and an HD95 of 5.02 mm. Testing on EchoNet-Dynamics demonstrates Dice 92.3% and HD95 4.05 mm. A comparative analysis with existing segmentation methods shows improvements in quantitative metrics. The AGSAM approach, as outlined by Zhou et al. [154], implements automatic segmentation in conditions of limited data using agent-based control of the SAM model. The architecture integrates an agent model based on FCN and DeepLabV3 [155] with pre-trained SAM Med2D [156], where the agent generates prompt embeddings to activate the SAM encoder and decoder. The FACM module augments features without increasing computational complexity. Experiments on the CAMUS and REFUGE [157] datasets demonstrate the approach’s effectiveness in few-shot scenarios. When trained on a single image, Dice metrics of 0.6829 for the endocardium, 0.5372 for the epicardium, and 0.5073 for the left atrial wall were achieved. The method has been shown to outperform comparable approaches, including nnSAM and AutoSAM, with a 5–12% advantage in Dice.

The nnSAM model proposed by Li et al. [158] integrates the SAM encoder with the nnUNet architecture for effective segmentation under limited data conditions. The dual-stream structure combines global features from ViT-SAM with local representations from nnUNet through concatenation [159]. On the BrainLes dataset for MRI white matter segmentation, Dice scores of 82.77% and ASD of 1.14 mm were achieved. For CT segmentation of heart structures on MM-WHS [160], the metrics were Dice 94.19% and ASD 1.36 mm. CT segmentation of the liver on Synapse demonstrates a Dice score of 85.24% and an ASD of 6.18 mm. From Montgomery County radiographic data, lung segmentation yielded a Dice 93.63% and an ASD of 1.47 mm. Subsequent studies have built on these foundations, offering further insights and extending the field’s scope. The CDSG-SAM [161] model is a cross-domain pipeline for brain tumour segmentation under limited data conditions, based on the SAM architecture. The method integrates the CDS adapter for multiscale feature extraction and the SG Prompt module for automatic prompt generation, thereby eliminating the need for manual input. The process was validated on three datasets: the BraTS2021 dataset [162] for glioma analysis, the CBTD clinical dataset for metastatic tumours, and the BraTS2023 dataset for both. Quantitative results show average Dice metrics of 0.918, 0.828, and 0.868, respectively.

Following the emergence of SAM adaptations with dual encoders, the MoE-SAM architecture introduces a Mixture-of-Experts structure to improve hierarchical feature utilization and automatic hint generation [163]. The MoE-SAM methodology consists of three main components: encoder adapters for finer tuning, a feature enhancement block (MoE-FEB) that integrates multi-level SAM representations, and a lightweight prompt embedding generator (LPEG) for autonomous prompt construction. An experimental evaluation of MoE-SAM across four medical datasets demonstrates consistent outperformance over SOTA methods. The quantitative findings show a Dice metric of 84.71% on Synapse CT, 89.38% on MMWHS, 76.82% on BTCV, and 91.89% on ACDC. In parallel, HD values of 8.756 mm, 13.67 mm, 5.637 mm, and 1.064 mm were recorded, respectively. Ablation analysis verifies the contribution of key components: integrating the MoE-FEB block yields a Dice gain of 1.46–1.53 percentage points.

Consequently, the CellSAM [164] methodology provided a fundamental framework for analysing cell images, integrating the Segment Anything Model with the CellFinder automated object detector. In this configuration, the general ViT encoder performs hierarchical feature extraction. In addition, two parallel modules are utilized: a bounding box detector and a SAM-based mask decoder. These modules are employed to delineate the individual cell structures [165]. This modification facilitates consistent segmentation of densely clustered nuclei. Table 3 below contains a comparative analysis of quantitative indicators of segmentation models based on the SAM architecture.

A comparative analysis of SAM adaptations reveals convergence towards hybrid architectures integrating specialised encoders with parametrically efficient tuning via LoRA mechanisms, achieving Dice scores of 81–95% while reducing annotation costs by 56–73% in weak supervision modes. The transition to volumetric segmentation expands the applicability of the paradigm, yet the need for domain adaptation remains.

## 10. Computational Efficiency and Parameter Optimization

The presence of parametric saturation in foundation models, along with associated inference delays, imposes significant computational constraints that impede the implementation of SAM architectures in clinical systems characterised by constrained hardware resources. In a recent study, Pu et al. [166] proposed the ClassWise-SAM-Adapter to improve computational efficiency by enabling parametrically efficient fine-tuning with a frozen SAM core. The architecture under discussion integrates lightweight adapters and a specialized class-wise mask decoder to transform instance segmentation into semantic segmentation. In addition, a task-specific input module was integrated, which injects low-frequency SAR features [167] via MLP layers [168]. This process compensates for the domain gap. Within this conceptual continuum, Trans-SAM [169] exemplifies the subsequent phase of parametrically efficient reconfiguration, implementing a dual-adapter mechanism that balances semantic preservation with alignment of spatial inductive bias. Trans-SAM implements a parametrically efficient adaptation strategy through two specialised modules. The intuitive perceptual adapter facilitates direct transfer of visual features from medical images to the SAM encoder, thereby preserving the semantic integrity of the original model. Concurrently, a multi-scale domain adapter incorporates spatial inductive biases.

In a recent paper, Asokan et al. [170] proposed a federated SAM tuning algorithm for volumetric medical segmentation that implements the principle of selective parametric updating via Low-Rank Adapters. When working with limited medical data, this methodology also identifies essential layers of the architecture. In a similar study, the LoRaMedNet framework [171] represents a key improvement in the field of effective adaptation of basic segmentation models for medical imaging. Going beyond the dependence on large-scale pre-training in this field, it rethinks generalization as a function of low-rank subspace reparameterization in the SAM encoder. And with limited fine-tuning and latent space modulation, the model preserves the semantic coherence of the base representation while enabling domain adaptation at minimal computational cost. This methodology redefines the balance between general and specialized optimization, demonstrating that structure-based LoRA optimization [172] can outperform fully retrained medical base models in both accuracy and efficiency. Table 4 summarizes representative parameter-efficient SAM frameworks and their key methodological innovations.

The evolution from full-parameter tuning to low-rank adaptation ensures the computational feasibility of SAM architectures for clinical deployment. However, it should be noted that systematic validation of the impact of parametric reduction on diagnostic reliability in heterogeneous medical modalities remains an understudied issue.

Practical implementation requires consideration of compatibility with PACS/DICOM conveyors and the ability to integrate SAM-based workflows into existing 3D-Slicer tool plugins. It is also important for clinical implementation to find a balance between interactive prompts and reducing the annotation workload.

## 11. Limitations and Future Directions

This section is devoted to the study of existing limitations and directions for further development of fundamental segmentation models based on the SAM architecture, with a focus on their application to medical images. The primary focus is on identifying methodological barriers that limit their generalization ability and computational efficiency. A significant limitation of SAM-based architectures in medical imaging is their reliance on substantial annotated datasets. Recent attempts to mitigate this limitation, exemplified by the Domain Tuning SAM framework [173], demonstrated that parameter-efficient tuning of the SAM encoder can reduce data requirements while preserving the structural accuracy of pre-trained representations. Wei et al. [174] presented the DAPSAM framework, which rethinks fine-tuning as a prototype-based generalization process. It uses a trainable memory bank to generate domain-adapted prototypes and integrates low-level semantic signals through hierarchical adapters. This framework reduces the over-specialization of SAM encoders observed in low-resource adaptation schemes. Wei et al. [174] also noted that the self-learning prompt generator creates a scalable mechanism for domain generalization from a single source.

It is imperative to acknowledge that addressing data scarcity requires implementing hybrid adaptation strategies that integrate parametrically efficient tuning with domain-specific learning. Nevertheless, reliance on a priori-defined architectures for out-of-distribution generalization remains an unresolved issue. The domain gap problem remains a key limitation, and even the universal pre-trained representation of SAM cannot fully account for specific visual artefacts. Ali et al. [175] noted that, despite SAM’s apparent success in transferring the concept of universal segmentation to the medical field, the results remain inconsistent. In particular, the model’s performance varies across modalities and structures.

## 12. Conclusions

This review examined the evolution of Segment Anything Model (SAM) architectures in medical imaging, which are developing along three directions: the adaptation of prompt mechanisms for clinical tasks, the integration of multimodal data, and the expansion of capabilities for 3D processing. Hybrid architectures with parametrically efficient LoRA tuning achieved Dice scores of 81–95% while concurrently reducing annotation costs by 56–73%. The transition to probabilistic methods facilitates the integration of uncertainty estimation into the segmentation process. The key challenges identified in this field include the following: sensitivity to inter-institutional data variations; dependence on large, labelled datasets; and the lack of standards for 3D models. The development of adaptive strategies for working with data from new sources is imperative in addressing these issues.

## Figures and Tables

**Table 1:** Prompt adaptation strategies for SAM in medical imaging.

Model	Prompt Method	Key Innovation	Domain	Ref.
AutoSAMUS	Automated prompt generation	End-to-end autonomous segmentation via auto-prompt generator	Ultrasound	[55]
LuGSAM	Text-driven prompts	Natural language descriptors converted to visual prompts	Chest radiography	[56]
Multi-text Joint Prompts	Decomposed semantic units	Clinical narratives broken into atomic units via cross-attention	Multi-modal image	[57]
YOLO + SAM	Detection-guided prompts	Automated spatial prompt generation for stable segmentation	Lung	[58]
PSAM	CLIP-controlled	Multimodal knowledge injection via semantic-visual adapter synergy	General medical imaging	[59]
DVPT	Dual local-global modulation	Fine-grained anatomical details via LFPT + noise suppression	Multi-scale medical structures	[60]
Med-SA	Hyper-Prompting mechanism	Task-specific conditioning with 13M and 600M trainable parameters	Domain transformation	[61]
U-Net to SAM	Two-stage prompt refinement	Classical segmentation converted to point prompts	Volumetric medical imaging	[62]
SAM-Path	Class-level trainable hints	Pathology-specific encoder	Whole-slide pathology	[63]
SAN	Auto-prompting support network	Thousands of point prompts	Histopathology	[65]
μSAM	Modality-specific retraining	Separate training for light and electron microscopy	Microscopic imaging	[66]
X-Gated Fusion	Hybrid architecture	U-Net local features + SAM global representations via gated attention	Nuclear structure segmentation	[67]
SegAnyPath	Multi-level adaptation	Multiscale proxy learning + Mixture of Experts architecture	Pathomorphological imaging	[68]
CellSAM	Asymmetric dual encoding	Cell-oriented with feature distillation	Cell microscopy	[73]
SAM-L	Molecular-driven learning	Weak annotations transformed via visual-biochemical marker alignment	Pathological structure annotation	[71]

**Table 2:** Uncertainty-aware SAM architectures for medical image segmentation.

Model	Uncertainty Method	Key Innovation	Ref.
SAM-U	Multi-box prompts + Monte Carlo sampling	Stochastic prediction space with ensemble-based variability assessment	[113]
I-MedSAM	Probabilistic prompt encoding	Latent space redefined as a stochastic mask generation domain	[109]
UR-SAM	Uncertainty-driven sampling + INR decoder	Continuous probability field via Implicit Neural Representation	[110]
E-BayesSAM	Ensemble prompts + adaptive redistribution	Uncertainty as an active self-correction mechanism	[118]
U-MedSAM	Token-wise variational Bayesian inference	Dynamic Bayesian estimation without retraining	[117]
EviPrompt	Sharpness-Aware Minimization (SharpMin)	Unified multi-scale confidence regulation	[115]
FNPC-SAM	Evidential reasoning + committee voting	Zero-shot prompt generation from a single reference pair	[119]
Cascaded Diffusion SAM	Multi-window augmentation + aleatory uncertainty	Error correction via probabilistic mask ensemble	[111]
P^2^SAM	Uncertainty-guided cross-attention	Mutual semantic field correction	[114]

**Table 3:** Quantitative Performance of SAM-based architectures in medical image segmentation (dice coefficient, %).

Model	Dataset	Dice Coefficient
SAM (Basic)	LGG	92.00
SMU-Net	Kvasir-SEG	94.60
SP-SAM	Kvasir-SEG	65.90
	ISIC 2018	66.90
PSF-SAM	Kvasir-SEG	93.93
	CVC-ClinicDB	94.21
FCSAM	Kvasir-SEG	88.10
BiSeg-SAM	Kvasir-SEG	90.90
	ETIS	90.40
SR-SAM	CVC-ClinicDB	81.46
	RUNMC	83.64
WSPolyp-SAM	Multiple medical datasets	90.70–94.50
LuGSAM	NIH CXR	97.00
MedCLIP-SAM	BUSI	85.60
	Brain Tumour MRI	88.10
	COVID-QU-Ex	91.30
TGSAM-2	ACDC	87.63
	MSD Spleen	89.34
	Prostate Ultrasound	92.75
	CVC-ClinicDB	85.10
AFTer-SAM	Thorax-85	92.75
	SegTHOR	92.42
AutoSAME	CAMUS	92.80
SAID-Net	CAMUS	93.20
	EchoNet-Dynamics	92.30
AGSAM	CAMUS (few-shot)	68.29 (endocardium)
		53.72 (epicardium)
		50.73 (left atrium)
nnSAM	BrainLes	82.77
	MM-WHS (heart CT)	94.19
	Synapse (Liver CT)	85.24
	Montgomery County	93.63
CDSG-SAM	BraTS2021	91.80
	CBTD	82.80
	BraTS2023	86.80
MoE-SAM	Synapse	84.71
	MMWHS	89.38
	BTCV	76.82
	ACDC	91.89

**Table 4:** Parameter-efficient adaptation strategies for SAM in medical imaging.

Model	Adaptation Method	Key Innovation	Reference
ClassWise-SAM-Adapter	Frozen SAM + lightweight adapters	Classwise mask decoder for instance-to-semantic transformation	[166]
Trans-SAM	Dual-adapter mechanism	Intuitive perceptual adapter + multi-scale domain adapter	[169]
Federated SAM	Low-Rank Adapters (LoRA) + selective layer tuning	Federated learning for volumetric segmentation	[170]
LoRaMedNet	Low-rank subspace reparameterization	Domain adaptation via latent space modulation	[171]

## Data Availability

No new data were created or analyzed in this study.
